# LncRNA GMDS‐AS1 inhibits lung adenocarcinoma development by regulating miR‐96‐5p/CYLD signaling

**DOI:** 10.1002/cam4.2776

**Published:** 2019-12-20

**Authors:** Ming Zhao, Xiao‐Feng Xin, Jian‐Ya Zhang, Wei Dai, Tang‐Feng Lv, Yong Song

**Affiliations:** ^1^ Department of Respiratory Medicine Jinling Hospital, Second Military Medical University Nanjing China

**Keywords:** CYLD, lncRNA GMDS‐AS1, lung adenocarcinoma, miR‐96‐5p

## Abstract

According to the global cancer statistic, lung cancer is one of the most dangerous tumors, which poses a serious threat to human health. Exploration the mechanism of lung cancer and new targeted therapeutic measures is always the hot topic. Long noncoding RNA (lncRNA) is an important factor affecting the development of tumors. However, the research on the mechanism of lncRNA in the progress of lung cancer needs to be further expanded. In this study, we found that the expression of lncRNA GMDS‐AS1 was significantly reduced in lung adenocarcinoma (LUAD) tissues and cells. Upregulated GMDS‐AS1 can significantly inhibit the proliferation of LUAD cells and promote cell apoptosis in vitro and in vivo. The results indicate that GMDS‐AS1 acts as a tumor suppressor gene to affect the development of LUAD. Further studies revealed that GMDS‐AS1 is a target gene of miR‐96‐5p, and GMDS‐AS1 regulates proliferation and apoptosis of LUAD cells in association with miR‐96‐5p. In addition, we also confirmed that CYLD lysine 63 deubiquitinase (CYLD) is also a target gene of miR‐96‐5p. Through various validations, we confirmed that GMDS‐AS1 can act as a ceRNA to upregulate the expression of CYLD by sponging miR‐96‐5p. Moreover, the intervention of GMDS‐AS1/miR‐96‐5p/CYLD network can regulate the proliferation and apoptosis of LUAD cells. In this study, we revealed that the GMDS‐AS1/miR‐96‐5p/CYLD network based on ceRNA mechanism plays an important role in the development of LUAD and provides a new direction and theoretical basis for targeted therapy of LUAD.

## Graphical abstract

GMDS‐AS1/miR‐96‐5p/CYLD network based on ceRNA mechanism plays an important role in the development of lung cancer and provides a new direction and theoretical basis for targeted therapy of lung cancer.

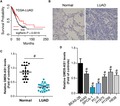

## 1 INTRODUCTION

Lung cancer is a malignant tumor originating from the bronchial mucosa or gland.^1^ About 700 000 people are diagnosed with lung cancer every year in China.^2^ The characteristics of easy metastasis, difficult surgical resection, and poor prognosis make it the most dangerous cancer, which leads to the highest mortality rate among all types of cancer.^3^ According to different histopathological features, it is divided into lung large cell carcinoma, lung squamous cell carcinoma, lung adenocarcinoma, lung adenosquamous carcinoma, and carcinoid.^4^ Among them, lung large cell carcinoma, lung squamous cell carcinoma, and lung adenocarcinoma belong to non–small‐cell lung cancer (NSCLC). In‐depth exploration of the pathogenesis mechanism of LUAD has become a top priority in current scientific research. Through basic research to find new targets for molecular therapy provides a new strategy for the diagnosis and prognosis of LUAD, and improves the survival rate and quality of life of patients with lung adenocarcinoma.

Noncoding RNAs refer to a class of RNA molecules that cannot be translated into proteins.^5^ They are mainly classified into short‐chain (including siRNA, miRNA, piRNA) and long‐length (long noncoding RNA, lncRNA) according to their length.^6^ LncRNA is a type of RNA which is longer than 200 in length and does not have the function of encoding protein.^7^ Initially, lncRNAs are thought to be RNAs that have no biologically function. However, subsequent studies have shown that lncRNAs play important roles in transcriptional, post‐transcriptional, and epigenetic processes.^8^ LncRNA has a structure similar to mRNA and has a promoter structure and a polyA tail.^9^ At present, it has been confirmed that lncRNA plays an important regulatory role in the proliferation, differentiation, development, and apoptosis of cells.^10^ In addition, lncRNA also plays a key role in the development of tumor cells. For example, lncRNA is involved in DNA methylation, chromatin remodeling, and genetic mechanisms of miRNAs, which can affect the development of tumors by disrupting the stability of the genome and affecting the function of the corresponding proteins.^11^ LncRNA LET is downregulated in cervical cancer,^12^ gastric cancer,^13^ and esophageal cancer,^14^ and its high expression predicts a good prognosis. In addition, lncRNA LET can inhibit the proliferation and EMT of lung adenocarcinoma cells.^15^ Downregulation of lncRNA LET promotes the proliferation and invasion of nasopharyngeal carcinoma cells.^16^ In addition, the same lncRNA may have different functions and mechanisms in different cancer cells. Both lncRNA HOTAIR and MALAT1 are revealed to be highly expressed in many tumor tissues, but they were downregulated in patients who are resistant to gefitinib.^17^, ^18^ LncRNA H19 plays an oncogenic role in NSCLC, and promotes tumor cell proliferation and migration,^19^ while acts as a tumor suppressor in thyroid cancer.^20^ Therefore, the function of lncRNA is not static and may be related to the intracellular environment. The exploration of the mechanism of lncRNA in tumorigenesis is still the tip of the iceberg and requires more attention and research. At present, no research has focused on the correlation between lncRNA GMDS‐AS1 and human diseases, and its mechanism in tumorigenesis and development is still unclear.

In this study, we demonstrate that GMDS‐AS1, as a tumor suppressor gene, is closely related to the development of LUAD. In LUAD cells, GMDS‐AS1 restrains cell proliferation and promotes cell apoptosis. Further studies have found that GMDS‐AS1 can act as a ceRNA to upregulate the expression level of CYLD by sponging miR‐96‐5p, which may be one of the mechanisms in LUAD. More importantly, in vivo study showed that GMDS‐AS1 can restrain the proliferation of LUAD cells. Our study reveals the mechanism of action of GMDS‐AS1 in the progress of LUAD and probes the application potential of LUAD‐targeted therapy.

## 2 MATERIALS AND METHODS

### 2.1 Cell culture and lung tissues

The cells used in this study included normal lung epithelial cells BEAS‐2B, and lung cancer cell line A549, SPCA‐1, PC‐9, H1975, H1299, and H838. These cells were purchased from the Cell Bank of Type Culture Collection of the Chinese Academy of Sciences. The cells were cultured in DMEM medium (HyClone), containing 10% FBS (Gibco), penicillin (100 U/mL), and streptomycin (100 mg/mL) at 37°C, 5% CO2.

LUAD tissues and paracancerous tissues were collected from Jinling Hospital of Nanjing. Patient specimens were obtained with informed consent, and the present study was approved by the Ethics Board of Jinling Hospital of Nanjing and was based on all relevant principles of the Declaration of Helsinki.

### 2.2 Plasmid, miRNA and cell transfection

The overexpression plasmids pcDNA‐GMDS‐AS1 and lentiviral overexpression plasmid Lenti‐GMDS‐AS1 were constructed by Sangon Company. Luciferase reporter plasmids include wild type and mutant pGLO‐GMDS‐AS1, as well as wild type and mutant pGLO‐CYLD 3'UTR, both constructed by Sangon Company. The mimic and inhibitor of miR‐96‐5p and CYLD siRNA were obtained from Genepharma Company; the sequence of miR‐96‐5p mimic was UUUGGCACUAGCACAUUUUUGCU, and the sequence of inhibitor was AGCAAAUUAGTCGUGTGCCAAA with the sequence of negative control of miR‐96‐5p was UUCUCCGAACGUGUCACGUUU. The plasmids or siRNAs were transfected using the Lipofectamine^®^ 2000 reagent (Invitrogen; Thermo Fisher Scientific, Inc). Cell transfection was performed as described previously.^21^

### 2.3 Reverse transcription and quantitative polymerase chain reaction (RT‐QPCR)

Tissues and cells were lysed by TRIzol reagent (Invitrogen), according to the manufacture instructions. RNA was extracted using the TRIzol^®^ reagent (Invitrogen; Thermo Fisher Scientific, Inc) in accordance with the manufacturer's protocol. RNA was subsequently reverse transcribed into complementary (c)DNA using a PrimeScript 1st Strand cDNA Synthesis Kit (TaKaRa). cDNA served as a template to detect the expression of the target gene using SYBR Premix Ex Taq II kit (Takara). The reverse transcription primer for miR‐96‐5p was GTCGTATCCAGTGCAGGGTCCGAGGTATTCGCACTGGATACGACCAGCAAA. The reverse transcription primer for U6 was GTCGTATCCAGTGCAGGGTCCGAGGTATTCGCACTGGATACGACAAATATG. The primers for qPCR included miR‐96‐5p (5ʹ‐TTTGGCACUAGCACATT‐3ʹ and 5ʹ‐GTGCAGGGTCCGAGGT‐3ʹ), U6 (5ʹ‐CTCGCTTCGGCAGCACA‐3ʹ and 5ʹ‐AACGCTTCACGAATTTGCGT‐3ʹ), GMDS‐AS1 (5ʹ‐AATGCTTTGAGGCCAAGCTA‐3ʹ and 5ʹ‐TGGGTTCATAAGGGTTGCAT‐3ʹ), CYLD (5ʹ‐TTGGCAACTGGGATGGAAGA‐3ʹ and 5ʹ‐TCCTTTCCTGCGTCACACTC‐3ʹ), CDKN2C (5ʹ‐GGGGACCTAGAGCAACTTACT‐3ʹ and 5ʹ‐CAGCGCAGTCCTTCCAAAT‐3ʹ, BRCA2 (5ʹ‐ACAAGCAACCCAAGTGTCAAT‐3ʹ and 5ʹ‐TGAAGCTACCTCCAAAACTGTG‐3ʹ, CDH1 (5ʹ‐ATTTTTCCCTCGACACCCGAT‐3ʹ and 5ʹ‐TCCCAGGCGTAGACCAAGA‐3ʹ), TP53 (5ʹ ‐GAGGTTGGCTCTGACTGTACC‐3ʹ and 5ʹ ‐TCCGTCCCAGTAGATTACCAC‐3ʹ), MAP2K4 (5ʹ‐TCCCAATCCTACAGGAGTTCAA‐3ʹ and 5ʹ‐CCAGTGTTGTTCAGGGGAGA‐3ʹ), and GAPDH (5ʹ‐CTGGGCTACACTGAGCACC‐3ʹ and 5ʹ‐AAGTGGTCGTTGAGGGCAATG‐3ʹ).

### 2.4 Western blot

RIPA lysis buffer (Biosharp, China) was used to extract protein from cells, and a bicinchoninic acid protein assay kit (Sangon, China) was used to quantify the concentration of protein obtained. Western blotting was performed as previously described.^21^ The antibodies used in this study include CYLD, cyclin A1, Bax, cleaved caspase‐3, PCNA, and GAPDH, which were obtained from Cell Signaling Technology.

### 2.5 Examination of cell proliferation

The cell proliferation was detected by colony formation assay and CCK‐8 assay. For the colony formation assay, cells of each group were seeded in a 6‐well plate at a density of 200 cells/well and cultured for 1 week. Cells were subsequently stained using crystal violet solution, after which the number of cell clones was counted. For the CCK‐8 assay, cells (3 × 10^3^ cells/well) in the logarithmic growth phase were cultured in 96‐well plates and incubated for 0 to 48 hours. After treatment, 10 µL CCK‐8 (Dojindo Molecular Technologies, Inc) was added and cells were further cultured for 2 hours. The OD450 value was measured using a microplate reader (BD Biosciences).

### 2.6 Cell apoptosis analysis

The effect of target genes on cell apoptosis was detected by flow cytometry. According to the manufacturer's protocol, the Annexin V‐FITC/PI Apoptosis Detection Kit (BD, USA) was used to label the cells, and then, labeled cells were analyzed by flow cytometry.

### 2.7 Luciferase assay

Luciferase assay was used to detect the effect of target genes on the activity of report gene plasmids. Cells (3 × 10^3^ cells/well) in the logarithmic growth phase were cultured in 96‐well plates and transfected using the Lipofectamine^®^ 2000 reagent (Invitrogen; Thermo Fisher Scientific, Inc). After transfection for 48 hours, Dual‐Luciferase reporter Assay Kit (Promega, USA) was used to detect the relative luciferase value in GloMax 20/20 Luminometer (Promega).

### 2.8 Nude mouse tumor model

Stable overexpressing cells were constructed in PC‐9 cells using lentiviral overexpression plasmids Lenti‐GMDS‐AS1 and Lenti‐NC. The nude mice were divided into two groups (10 mice each group). 1 × 10^7^ stably overexpressing PC‐9 cells were injected subcutaneously into the back of nude mice, and the growth of the back ticks in nude mice was observed and recorded every three days. When the neoplasm grows to a suitable size, the nude mice are sacrificed by cervical dislocation, and the neoplasm is removed, and the volume and weight of the scorpion are counted and stored in liquid nitrogen. Our study was approved by the Ethics Board of Jinling Hospital of Nanjing.

### 2.9 Statistical analysis

All experiments were repeated at least three times, and representative images were presented. A one‐way ANOVA method was used for comparisons between two groups. *P* < .05 was considered to indicate a statistically significant difference.

## 3 RESULTS

### 3.1 GMDS‐AS1 is downregulated in the progress of LUAD and positively correlated with survival of LUAD patients

**Figure 1:**
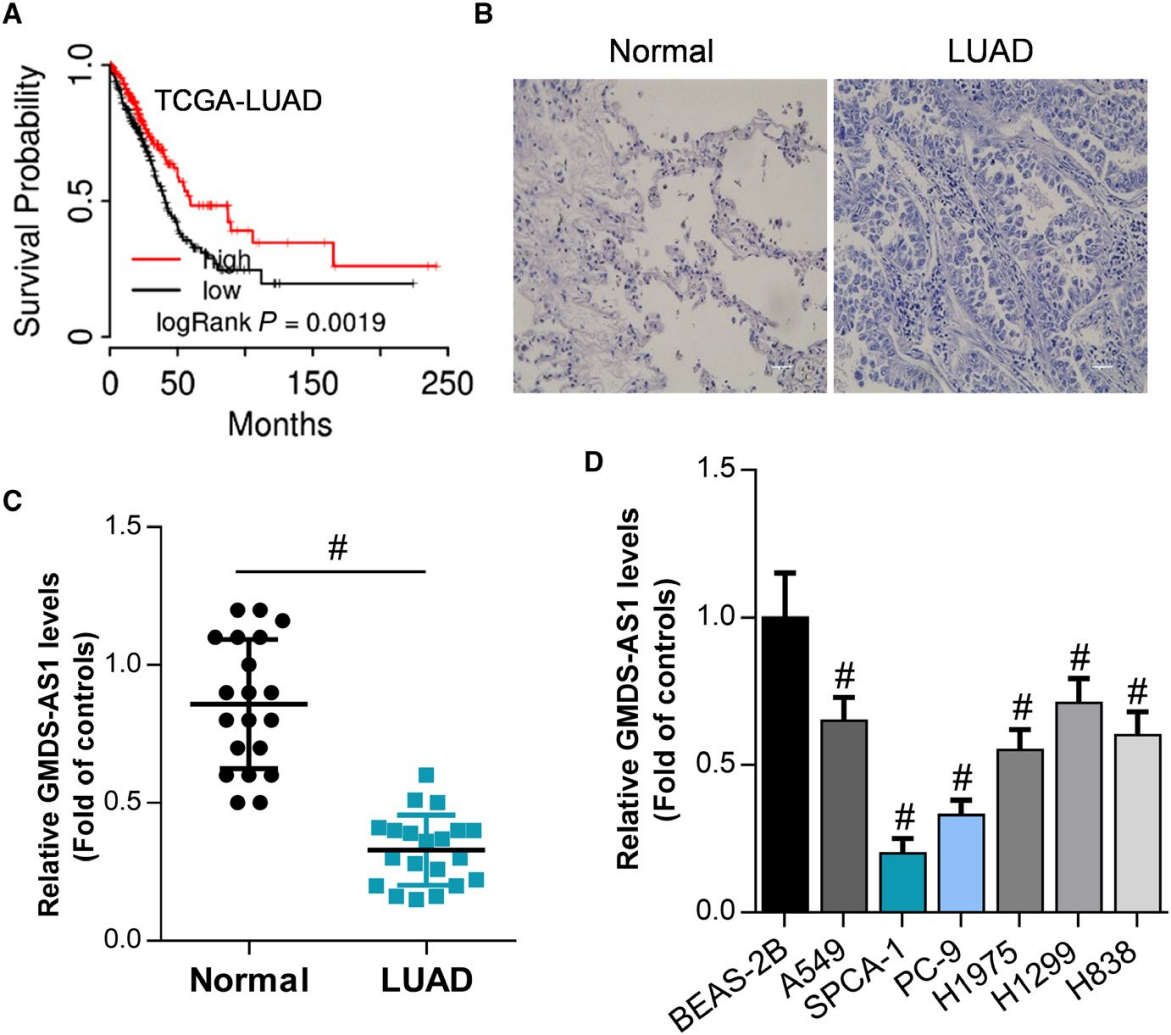
GMDS‐AS1 is downregulated in lung cancer tissues and cells. (A) Analysis of the correlation between GMDS‐AS1 and prognosis of LUAD patients by bioinformatics. We collected 20 cases of LUAD tissues and paracancerous tissues (B) and detected mRNA expression levels of GMDS‐AS1 in LUAD tissues and adjacent tissues by RT‐qPCR (C), ^#^ *P* < .05, compared with adjacent controls. (D) In normal lung epithelial cells BEAS‐2B, and lung cancer cells A549, SPCA‐1, PC‐9, H1975, H1299, and H838, the expression level of GMDS‐AS1 was detected by RT‐qPCR, ^#^ *P* < .05, compared with BEAS‐2B

There are currently no studies showing differential expression of GMDS‐AS1 in LUAD and adjacent tissues. Through bioinformatics analysis, we found that higher expression of GMDS‐AS1 predicted the longer survival of patients with LUAD (Figure 1A). Then, we collected 20 pairs of LUAD tissues and their adjacent tissues (Figure 1B). The results of RT‐qPCR showed that the expression level of GMDS‐AS1 was significantly downregulated in LUAD tissues (Figure 1C). In addition, the expression levels of GMDS‐AS1 in lung cancer cells A549, SPCA‐1, PC‐9, H1975, H1299, and H838 cells were also significantly reduced relative to normal lung epithelial cells BEAS‐2B (Figure 1D). Furthermore, we also confirmed that the expression of GMDS‐AS1 was positively related to the histological grade of LUAD while negatively related to the tumor size (Table 1). These results suggest that GMDS‐AS1 is downregulated in lung cancer, which was closely related to the development of lung cancer.

**Table 1:** The clinic‐pathological factors of 20 NSCLC patients

Characteristics	Numbers	Expression of GMDS‐AS1		*P* value
		Low (N = 10)	High (N = 10)	
Sex				.431
Male	9	5	4	
Female	11	5	6	
Age				.718
≤60	10	5	5	
＞60	10	5	5	
Histological grade				.025
Middle or low	14	5	9	
High	6	5	1	
Histological classification				.611
Squamous cell carcinoma	9	4	5	
Adenocarcinoma or other	11	5	7	
TNM Stage				.101
I and II	14	6	8	
III and IV	6	4	2	
Tumor size				.012
≤3 cm	7	5	2	
＞3 cm	13	5	8	
History of smoking				.207
Ever	11	5	6	
Never	9	4	4	

### 3.2 GMDS‐AS1 can restrain the proliferation of LUAD cells while induce cell apoptosis in vitro and in vivo

**Figure 2:**
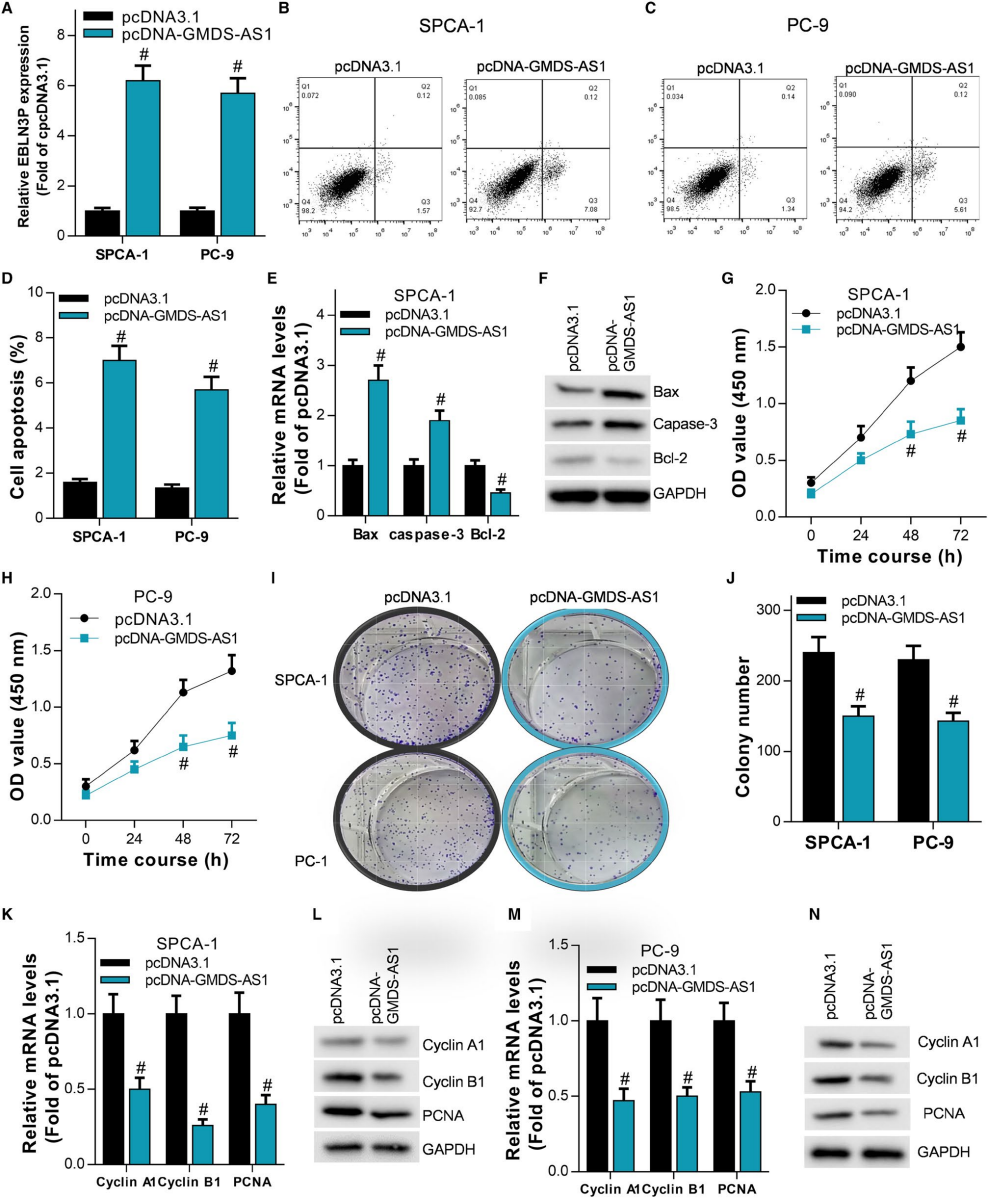
GMDS‐AS1 inhibits LUAD cell proliferation and promotes apoptosis in vitro. We transfected the overexpression plasmid pcDNA‐GMDS‐AS1 and the negative control pcDNA3.1 into SPCA‐1 and PC‐9 cells, and detected the expression level of GMDS‐AS1 by RT‐qPCR (A), ^#^ *P* < .05, compared with pcDNA3.1. (B, C, D) The apoptosis of SPCA‐1 and PC‐9 cells was detected by flow cytometry, ^#^ *P* < .05, compared with pcDNA3.1. (E, F) The mRNA and protein expression levels of Bax, caspase‐3, and Bcl‐2 were detected by RT‐qPCR and Western blot, ^#^ *P* < .05, compared with pcDNA3.1. The proliferation ability of LUAD cells SPCA‐1 and PC‐9 cells was examined by CCK‐8 assay (G, H) and colony formation assay (I, J), ^#^ *P* < .05, compared with pcDNA3.1. (K‐N) The mRNA expression levels of cyclin A1, cyclin B1 and PCNA were detected by RT‐qPCR and Western blot in SPCA‐1 and PC‐9 cells, ^#^ *P* < .05, compared with pcDNA3.1

**Figure 3:**
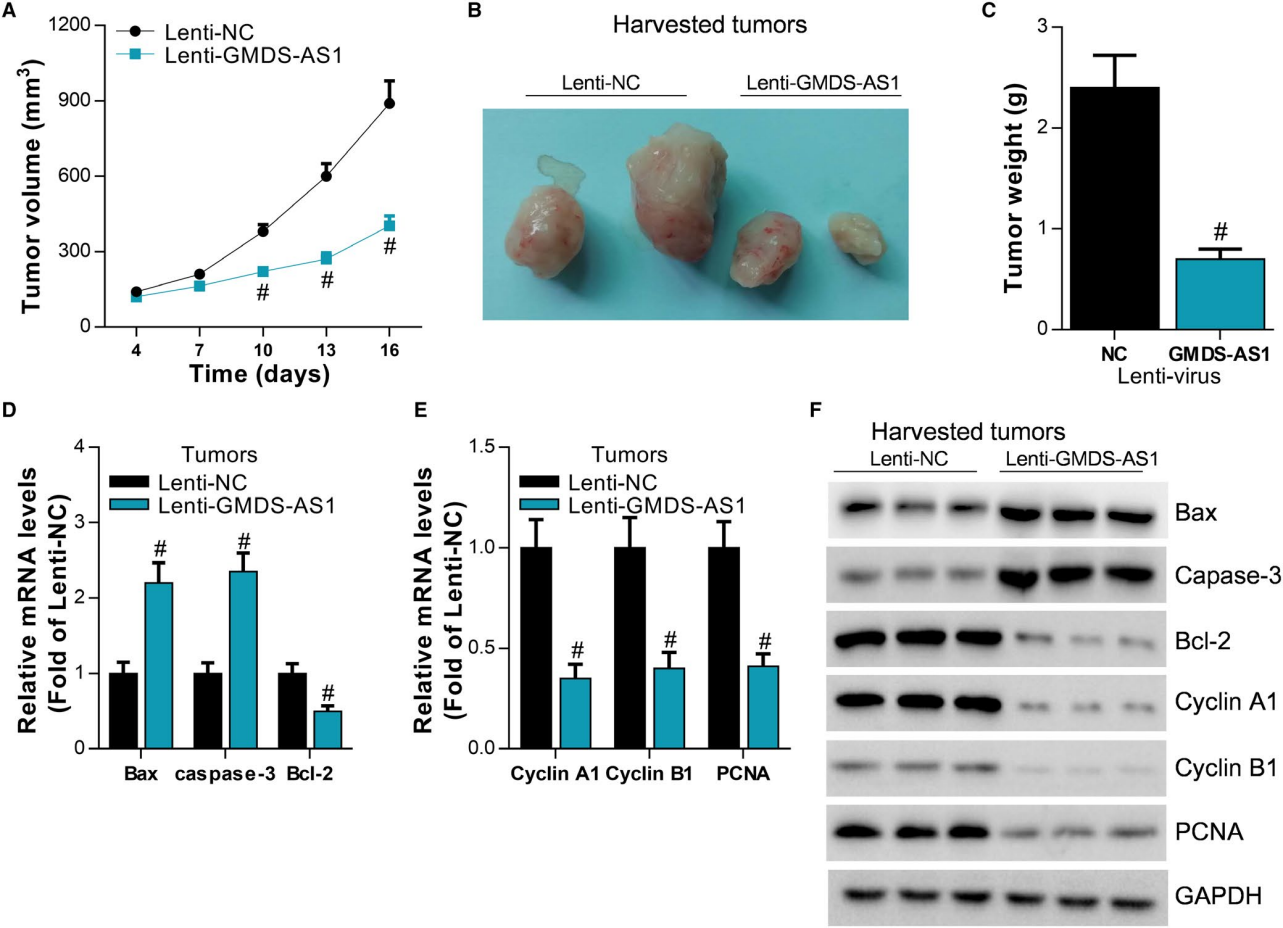
GMDS‐AS1 inhibits LUAD cell proliferation in vivo. (A, B, C) PC‐9 cells stably overexpressing GMDS‐AS1 were injected subcutaneously into nude mice, and the growth of neoplasms was monitored in real time. After the nude mice were sacrificed, the neoplasms were removed and the weight was counted, ^#^ *P* < .05, compared with Lenti‐NC. (D, E) The expression levels of Bax, caspase‐3, Bcl‐2, cyclin A1, cyclin B1, and PCNA in neoplasms were detected by RT‐qPCR and Western blot, ^#^ *P* < .05, compared with Lenti‐NC

We have observed that GMDS‐AS1 is downregulated in LUAD tissues and cells. To observe the effect of GMDS‐AS1 on the biological behavior of LUAD cells, we constructed the overexpression plasmid pcDNA‐GMDS‐AS1 and pcDNA3.1 as a negative control. SPCA‐1 and PC‐9 cells were transfected with pcDNA‐GMDS‐AS1 and pcDNA3.1 (Figure 2A), and cell apoptosis was detected by flow cytometry and the cell proliferation ability was observed by CCK‐8 assay and colony formation assay. In SPCA‐1 and PC‐9 cells, flow cytometry results showed that overexpression of GMDS‐AS1 significantly promoted cell apoptosis (Figure 2B‐D). Bax, caspase‐3 and Bcl‐2 are both apoptosis‐related proteins. The increased expression levels of Bax and caspase‐3 can promote apoptosis, while Bcl‐2 can inhibit apoptosis.^22^ RT‐qPCR results showed that overexpression of GMDS‐AS1 significantly upregulated the expression levels of Bax and caspase‐3 and inhibited the expression of Bcl‐2 (Figure 2E,F). The results of the CCK‐8 assay showed that overexpression of GMDS‐AS1 significantly inhibited the proliferation of cells (Figure 2G,H), which were consistent with the results of colony formation assay (Figure 2I,J). Cyclin A1 and cyclin B1 belong to the cyclin family, which accelerate the cell cycle by interacting with cyclin‐dependent kinases (CDKs).^23^ Therefore, upregulation of cyclin A1 and cyclin B1 expression levels can promote cell proliferation. PCNA is also used to measure the ability of cell proliferation, and its upregulation can promote cell proliferation. In SPCA‐1 and PC‐9 cells, the results of RT‐qPCR showed that overexpression of GMDS‐AS1 significantly inhibited the mRNA expression levels of cyclin A1, cyclin B1, and PCNA (Figure 2K‐N), which is consistent with the results of the CCK‐8 assay and the clone formation assay. Furthermore, we observed the effect of GMDS‐AS1 on LUAD cells in vivo. We found that overexpression of GMDS‐AS1 significantly inhibited the growth rate and weight of LUAD neoplasms (Figure 3A‐C). Moreover, RT‐qPCR results also showed that the expression levels of Bax and caspase‐3 were significantly increased, while the expression levels of cyclin A1, cyclin B1, PCNA, and Bcl‐2 were significantly decreased in the GMDS‐AS1 overexpression group (Figure 3D‐F), which was consistent with the in vitro study. These results indicate that GMDS‐AS1 acts as a tumor suppressor gene and plays an important role in the development of LUAD.

### 3.3 GMDS‐AS1 is the target gene of MIR‐96‐5p

**Figure 4:**
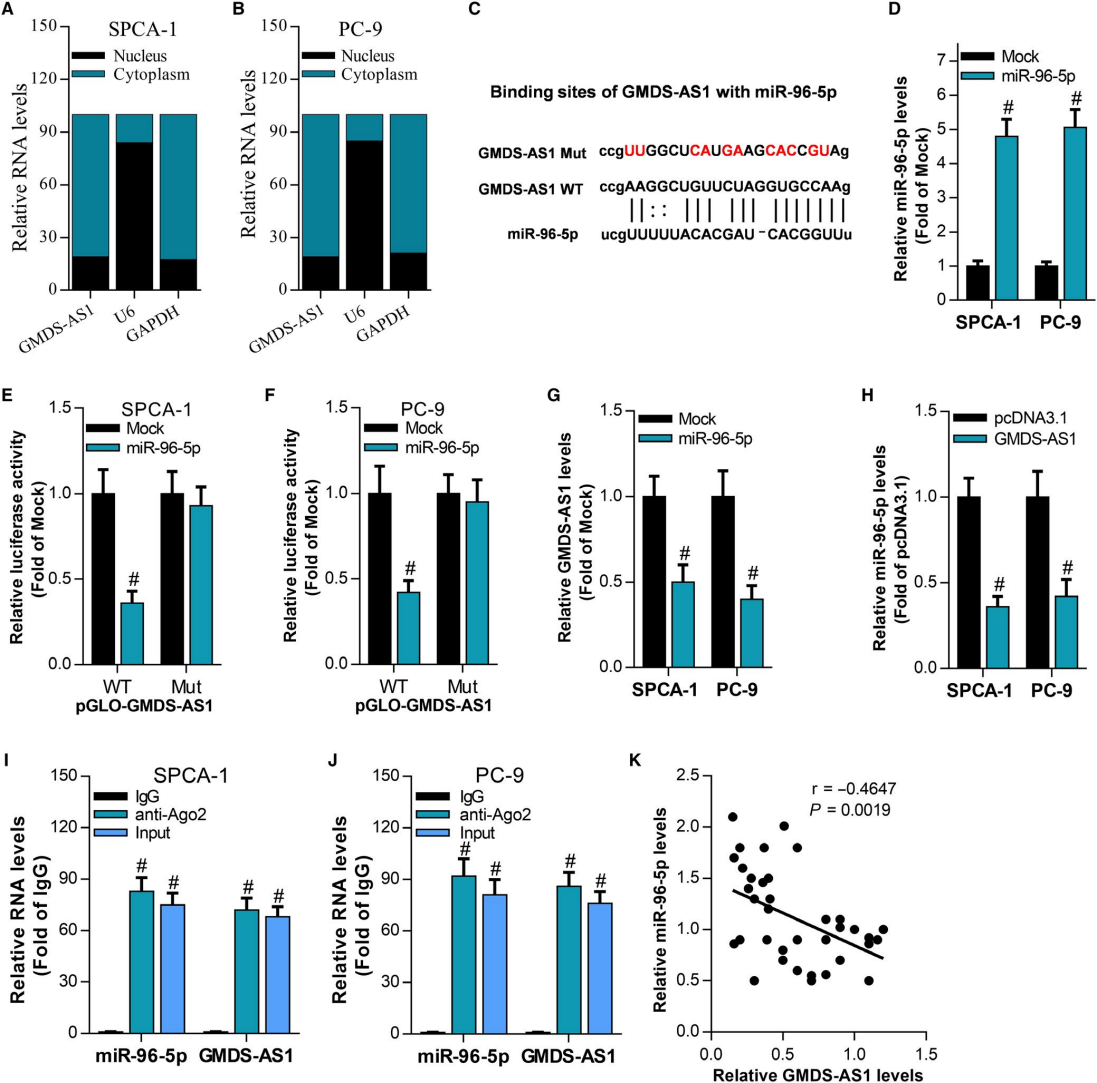
GMDS‐AS1 is the target gene of miR‐96‐5p. (A, B) The expression of GMDS‐AS1 in cytoplasm and nucleus was separately detected by RT‐qPCR in SPCA‐1 and PC‐9 cells. (C) Prediction of interaction of miR‐96‐5p with GMDS‐AS1 by bioinformatics and mutation of the binding site. miR‐96‐5p mimic (miR‐96‐5p) and negative control Mock were transfected into SPCA‐1 and PC‐9 cells, and (D) the expression level of miR‐96‐5p was detected by RT‐qPCR, ^#^ *P* < .05, compared with Mock. (E, F) The effect of miR‐96‐5p on the activity of pGLO‐GMDS‐AS1 WT and pGLO‐GMDS‐AS1 Mut was examined by luciferase assay, ^#^ *P* < .05, compared with Mock. (G, H) In SPCA‐1 and PC‐9 cells, the regulation between miR‐96‐5p and GMDS‐AS1 expression was detected by RT‐qPCR, ^#^ *P* < .05, compared with Mock or pcDNA3.1. (I, J) The interaction of miR‐96‐5p and GMDS‐AS1 was detected by Ago2 RIP‐qPCR assay, ^#^ *P* < .05, compared with IgG. (K) The relationship of GMDS‐AS1 expression and miR‐96‐5p expression was analyzed by RT‐qPCR using Pearson correlation analysis

There was no study that showed the location of GMDS‐AS1 in the cells. In this study, we separately extracted the RNA in cytoplasm or nucleus. The results of qPCR confirmed that GMDS‐AS1 mainly located in the cytoplasm (Figure 4A,B). Through bioinformatics prediction, we found that GMDS‐AS1 contains the binding sequence of miR‐96‐5p. Based on the predicted binding sites, we designed the GMDS‐AS1 reporter plasmid pGLO‐GMDS‐AS1 containing the wild‐type binding sequence (WT) and the mutant binding sequence (Mut), respectively (Figure 4C). To verify whether GMDS‐AS1 is a target gene for miR‐96‐5p, we synthesized miR‐96‐5p mimic (miR‐96‐5p) (Figure 4D). In SPCA‐1 and PC‐9 cells, luciferase assay showed that miR‐96‐5p significantly inhibited the activity of the pGLO‐GMDS‐AS1 WT reporter gene, but had no significant effect on the pGLO‐GMDS‐AS1 Mut (Figure 4E,F). In addition, miR‐96‐5p significantly inhibited the expression level of GMDS‐AS1 in SPCA‐1 and PC‐9 cells. Conversely, GMDS‐AS1 also inhibited the expression level of miR‐96‐5p (Figure 4G,H). The miRNA forms an RNA‐inducing silencing complex with the Dicer enzyme and the Ago 2 protein, thereby binding and inhibiting the expression of the target gene. Therefore, if GMDS‐AS1 is a target gene of miR‐96‐5p, the RNA‐induced silencing complex formed by miR‐96‐5p and Dicer enzyme, Ago 2 protein can bind to GMDS‐AS1. miR‐96‐5p and GMDS‐AS1 were simultaneously detected by RIP‐qPCR assay of Ago 2 protein. As we predicted, the results of the RIP‐qPCR experiment showed that miR‐96‐5p may interact with GMDS‐AS1 (Figure 4I, J). Furthermore, we also confirmed the expression of miR‐96‐5p was negatively related to GMDS‐AS1 in LUAD (Figure 4K). These findings reveal that miR‐96‐5p directly targets to GMDS‐AS1.

### 3.4 MIR‐96‐5p promotes cell proliferation in LUAD by targeting CYLD

**Figure 5:**
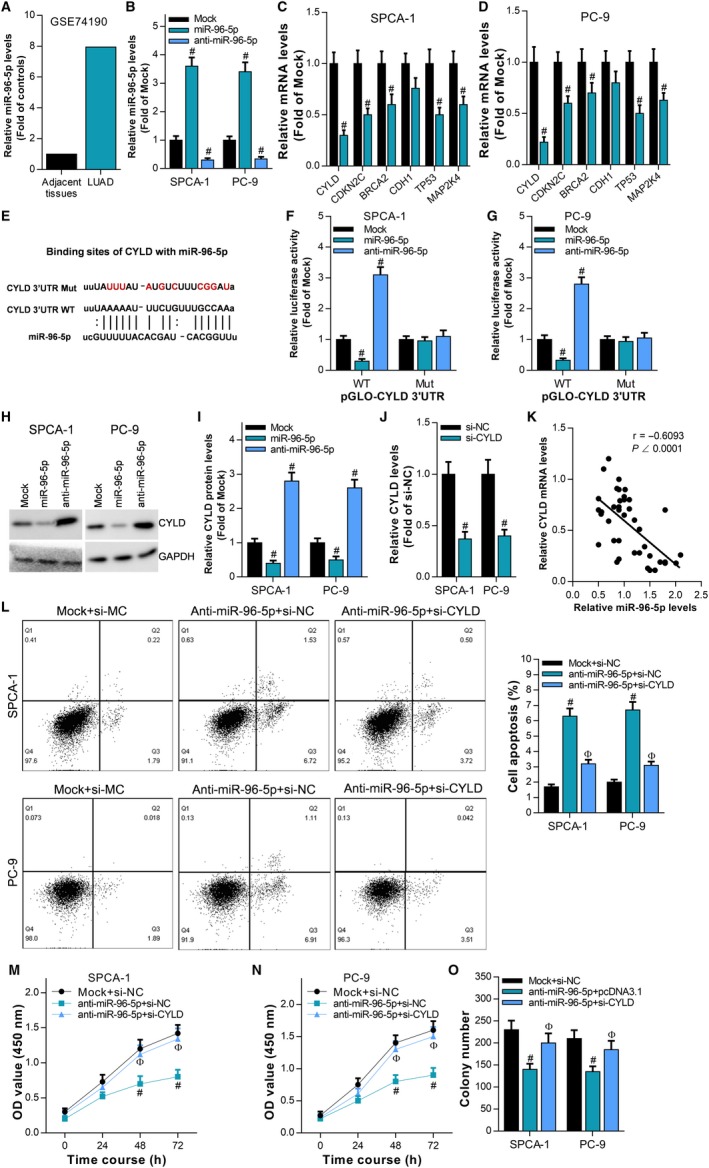
miR‐96‐5p promotes proliferation of LUAD cells by targeting CYLD. A, Analysis of miR‐96‐5p expression in LUAD tissues and adjacent tissues by transcriptome sequencing data. miR‐96‐5p mimic (miR‐96‐5p) and miR‐96‐5p inhibitor (anti‐miR‐96‐5p) were transfected into SPCA‐1 and PC‐9 cells, respectively. B, RT‐qPCR was used to detect the expression level of miR‐96‐5p, ^#^ *P* < .05, compared with Mock. C, D, The effect of miR‐96‐5p on the expression levels of CYLD, CDKN2C, BRCA2, CDH1, TP53, and MAP2K4 was detected by RT‐qPCR, ^#^ *P* < .05, compared with Mock. E, Predicting the interaction of miR‐96‐5p with CYLD 3'UTR by bioinformatics, and mutating the binding site to generate the reporter plasmids pGLO‐CYLD 3'UTR WT and pGLO‐CYLD 3 'UTR Mut. F, G, The effect of miR‐96‐5p on the activity of pGLO‐CYLD 3' UTR WT and pGLO‐CYLD 3' UTR Mut was examined by luciferase assay in SPCA‐1 and PC‐9 cells, ^#^ *P* < .05, compared with Mock. H, I, The effects of miR‐96‐5p and anti‐miR‐96‐5p on the expression of CYLD protein were detected by Western blot in SPCA‐1 and PC‐9 cells, ^#^ *P* < .05, compared with Mock. J, CYLD siRNA (si‐CYLD) was transfected into SPCA‐1 and PC‐9 cells, and the expression level of CYLD was detected by RT‐qPCR, ^#^ *P* < .05, compared with si‐NC. K, The relationship of miR‐96‐5p expression and CYLD expression was analyzed by RT‐qPCR using Pearson correlation analysis. L, The effect of anti‐miR‐96‐5p and/or si‐CYLD on cell apoptosis was examined by flow cytometry, ^#^ *P* < .05, compared with Mock + si‐NC. ^Ф^ *P* < .05, compared with anti‐miR‐96‐5p + si‐NC. The effect of anti‐miR‐96‐5p and/or si‐CYLD on cell proliferation was examined by CCK‐8 assay (M, N) and colony formation assay (O), ^#^ *P* < .05, compared with Mock + si‐NC. ^Ф^ *P* < .05, compared with anti‐miR‐96‐5p + si‐NC

Through bioinformation analysis, we found that miR‐96‐5p was significantly increased in LUAD tissues (Figure 5A). Then, we synthesized miR‐96‐5p mimic (miR‐96‐5p) and inhibitor (anti‐miR‐96‐5p) and transfected them into SPCA‐1 and PC‐9 cells (Figure 5B). RT‐qPCR results showed that miR‐96‐5p significantly inhibited the expression levels of CYLD, CDKN2C, BRCA2, CDH1, TP53, and MAP2K4. Among them, miR‐96‐5p has the most obvious inhibitory effect on CYLD (Figure 5C,D). Based on the predicted binding sequences, we constructed reporter plasmids pGLO‐CYLD 3'UTR containing the wild‐type binding sequence (WT) and the mutant binding sequence (Mut), respectively (Figure 5E). In SPCA‐1 and PC‐9 cells, the activity of pGLO‐CYLD 3'UTR WT was inhibited by miR‐96‐5p, while activated by anti‐miR‐96‐5p, but miR‐96‐5p and anti‐miR‐96‐5p both have no effect on the activity of pGLO‐CYLD 3'UTR Mut (Figure 5F,G). Western blot results indicated that the expression of CYLD was significantly inhibited by miR‐96‐5p, while promoted by anti‐miR‐96‐5p (Figure 5H,I). CYLD siRNA (si‐CYLD) was synthesized and then cotransfected with anti‐miR‐96‐5p into SPCA‐1 and PC‐9 cells (Figure 5J). Furthermore, we also confirmed the expression of miR‐96‐5p was negatively related to CYLD in LUAD (Figure 5K). Flow cytometry results showed that si‐CYLD could reverse the effect of anti‐miR‐96‐5p on apoptosis of LUAD cells (Figure 5L). The results of CCK‐8 assay (Figure 5M,N) and colony formation assay (Figure 5O) showed that si‐CYLD could reverse the inhibitory effect of anti‐miR‐96‐5p on the proliferation of LUAD cells. These results indicate that miR‐96‐5p enhances the cell proliferation ability in LUAD by targeting CYLD.

### 3.5 GMDS‐AS1/MIR‐96‐5p/CYLD network based on ceRNA mechanism regulates proliferation and apoptosis of LUAD cells

**Figure 6:**
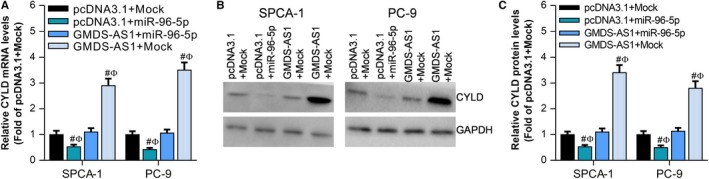
GMDS‐AS1 acts as a ceRNA to upregulate the expression of CYLD by sponging miR‐96‐5p. In SPCA‐1 and PC‐9 cells, (A) the effect of GMDS‐AS1 overexpression and/or miR‐96‐5p on CYLD mRNA expression was examined by RT‐qPCR. (B, C) The effect of GMDS‐AS1 overexpression and/or miR‐96‐5p on CYLD protein expression was detected by Western blot. ^#^ *P* < .05, compared with pcDNA3.1 + Mock. ^Ф^ *P* < .05, compared with GMDS‐AS1 + miR‐96‐5p

We have demonstrated that both GMDS‐AS1 and CYLD are target genes of miR‐96‐5p. Can GMDS‐AS1 act as a ceRNA to regulate the expression of CYLD by sponging miR‐96‐5p? In SPCA‐1 and PC‐9 cells, RT‐qPCR results showed that miR‐96‐5p can inhibit the expression of CYLD mRNA, while overexpression of GMDS‐AS1 can reverse the inhibitory effect of miR‐96‐5p on CYLD expression (Figure 6A). The results of Western blot were consistent with the results of RT‐qPCR (Figure 6B,C). These results indicate that GMDS‐AS1 can act as a ceRNA to upregulate the expression of CYLD by sponging miR‐96‐5p.

**Figure 7:**
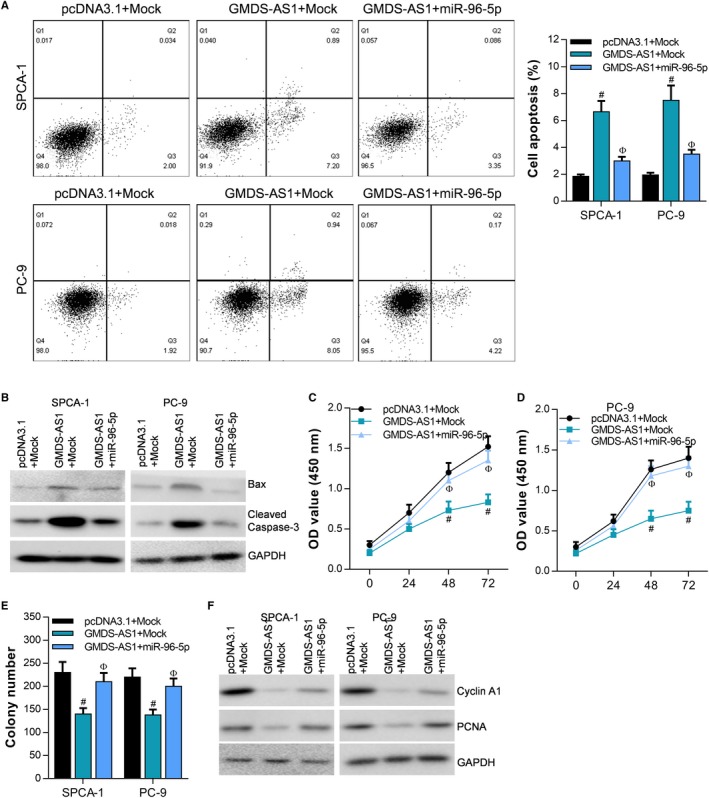
GMDS‐AS1/miR‐96‐5p/CYLD network regulates the proliferation and apoptosis of LUAD cells. In SPCA‐1 and PC‐9 cells, (A) the effect of GMDS‐AS1 overexpression and/or miR‐96‐5p on cell apoptosis was examined by flow cytometry. (B) The effect of GMDS‐AS1 overexpression and/or miR‐96‐5p on the expression of Bax and cleaved caspase‐3 was detected by Western blot. The effect of GMDS‐AS1 overexpression and/or miR‐96‐5p on cell proliferation was examined by CCK‐8 assay (C, D) and colony formation assay (E). (F) The effect of GMDS‐AS1 overexpression and/or miR‐96‐5p on the expression of cyclin A1 and PCNA was detected by Western blot. ^#^ *P* < .05, compared with pcDNA3.1 + Mock. ^Ф^ *P* < .05, compared with GMDS‐AS1 + Mock

In SPCA‐1 and PC‐9 cells, miR‐96‐5p can inhibit the effect of GMDS‐AS1 on cell apoptosis (Figure 7A), and the results of Western blot also show that GMDS‐AS1 can upregulate the apoptosis‐related proteins Bax and cleaved caspase‐3, while miR‐96‐5p can reverse the effect of GMDS‐AS1 (Figure 7B). In addition, CCK‐8 assay (Figure 7C,D) and colony formation assay (Figure 7E) showed that miR‐96‐5p can reverse the effect of GMDS‐AS1 on cell proliferation. The results of Western blot also show that GMDS‐AS1 can inhibit the expression of cyclin A1 and PCNA, while miR‐96‐5p can reverse the effect of GMDS‐AS1 (Figure 7F). These findings suggest that the GMDS‐AS1/miR‐96‐5p/CYLD network based on the ceRNA mechanism can regulate the proliferation and apoptosis of LUAD cells.

## 4 DISCUSSION

It is currently thought that there are four mechanisms by which lncRNAs regulate gene expression. Firstly, the expression of genes is regulated at the epigenetic level. LncRNA acts as a platform to recruit epigenetic modifiers for cis‐ or trans‐regulation. For example, lncRNA HOTAIR and HOTTIP can recruit chromosome modifiers PRC2 and MLL to specific sites for genome modification.^24^, ^25^ Secondly, processing or degrading mRNA. Every step of RNA metabolism is regulated precisely and complexly. Recently, it has been reported that lncRNA can control the stability of RNA and the processing of mRNA precursors. An example of lncRNA regulating mRNA precursor processing is a nuclear localized lncRNA MALAT1, in which MALAT1 regulates selective cleavage of mRNA by assembling a serine/arginine cleavage factor in the nucleus.^26^ Thirdly, post‐transcriptional level regulation. LncRNAs, as competitive endogenous RNAs (ceRNAs), are capable of regulating the expression of protein‐coding genes by their miRNA binding sites similar to the 3' UTR region of mRNA. In lung cancer cells, lncRNA LINC‐PINT can act as a ceRNA to regulate the expression of PDCD4 by sponging miR‐208a‐3p.^27^ LncRNA EPB41L4A‐AS2 acts as an efficient sponge of miR‐301a‐5p, reversing the inhibitory effect of miR‐301a‐5p on FOXL1 expression.^28^ Fourthly, regulation of the protein activity. LncRNAs are capable of binding proteins and regulating their activity. For example, lncRNA Evf‐2 can bind to the transcription factors Dlx/Dll to promote transcription of downstream genes.^29^ In addition, lncRNA PRNCR1 and PCGEM1 are capable of binding to androgen receptors in prostate cancer to synergistically regulate the transcription of target genes.^30^

The key role of lncRNA in the development of tumors is unquestionable. In the present study, we found that lncRNA GMDS‐AS1 is downregulated in the development of LUAD, whereas overexpression of GMDS‐AS1 can inhibit the proliferation of LUAD cells and promote apoptosis. These results indicate that GMDS‐AS1 is a tumor suppressor in LUAD. The ceRNA mechanism is one of the most classical research directions of the mechanism of lncRNA. It is well known that miRNAs bind to the 5’UTR or 3’UTR region and silence or degrade the mRNA.^31^ Previous studies have suggested that miRNA mainly targets mRNA. However, with the advent and deep research of lncRNA, it was found that lncRNA has a structure similar to mRNA.^32^ Therefore, lncRNA can also act as a target gene of miRNAs. These miRNA response elements (MREs) are the structural basis for the function of miRNAs, while noncoding RNAs can also interact with miRNAs through MREs, which makes ceRNAs have the potential to regulate gene expression. The regulation mechanism of ceRNA is reflected in the fact that ceRNAs with the similar MREs reverse the inhibition of the miRNA on mRNA by competitively binding miRNAs. The ceRNAs compete for binding to a common miRNA to achieve mutual regulation, which is called the ceRNA mechanism. LncRNA plays a key role in the development of lung cancer through the ceRNA mechanism. For example, lncRNA LINC‐PINT can act as a ceRNA to promote the expression of PDCD4 by sponging miR‐208a‐3p, thereby inhibiting the proliferation and migration of lung cancer cells.^27^ LncRNA DANCI acts as an efficient sponge of miR‐138 and reverses the inhibitory effect of miR‐138 on Sox4 expression, which enhances the proliferation and metastasis ability of lung cancer cells.^33^ In this study, we found through bioinformatics analysis that both GMDS‐AS1 and CYLD contain the binding sequence of miR‐96‐5p. Further studies revealed that miR‐96‐5p can inhibit the expression of GMDS‐AS1 and CYLD. These results indicate that both GMDS‐AS1 and CYLD are target genes of miR‐96‐5p. More importantly, overexpression of GMDS‐AS1 reversed the inhibitory effect of miR‐96‐5p on CYLD. Therefore, our study revealed the mechanism by which GMDS‐AS1 acts as a ceRNA to upregulate CYLD expression by sponging miR‐96‐5p. In addition, our study confirmed that GMDS‐AS1 overexpression inhibits the proliferation of LUAD cells in nude mice. miR‐96‐5p reverses the effect of GMDS‐AS1 on proliferation and apoptosis of LUAD cells, suggesting that GMDS‐AS1/miR‐96‐5p/CYLD network plays a key role in the development of LUAD (Figure 7).

In this study, we revealed that GMDS‐AS1 acts as an oncogene in LUAD. In LUAD cells, GMDS‐AS1 acts as a ceRNA, which promotes the expression of CYLD by sponging miR‐96‐5p, thereby inhibiting the proliferation of tumor cells and promoting apoptosis. Our study reveals the important role of the ceRNA mechanism‐based GMDS‐AS1/miR‐96‐5p/CYLD network in the development of LUAD and provides a new strategy for targeted therapy of LUAD.

## CONFLICT OF INTEREST

All authors declare that there are no conflicts of interests.

## Data Availability

The data used to support the findings of this study are available from the corresponding author upon request.
